# Sulfosuccinimidyl oleate ameliorates the high-fat diet-induced obesity syndrome by reducing intestinal and hepatic absorption

**DOI:** 10.3389/fphar.2023.1193006

**Published:** 2023-05-26

**Authors:** Qiming Ma, Li Wen, Yanxia Tian, Liqin Ma, Zhangsheng Wen, Yang Kun, Mengping Xu, Xiaoping Liu

**Affiliations:** ^1^ The Department of General Surgery, The First Affiliated Hospital of Gannan Medical University, Ganzhou, Jiangxi, China; ^2^ Department of Neonatology, Ganzhou Maternal and Child Health Centre, Ganzhou, Jiangxi, China; ^3^ Department of Blood Transfusion, The First Affiliated Hospital of Gannan Medical University, Ganzhou, Jiangxi, China; ^4^ The Second Department of Surgery, People’s Hospital of Shicheng County, Ganzhou, Jiangxi, China; ^5^ The CT Room of the Imaging Department, People’s Hospital of Shicheng County, Ganzhou, Jiangxi, China

**Keywords:** intestine, obesity, lipid absorption, sulfosuccinimidyl oleate, high-fat diet

## Abstract

**Background:** A high-fat Western diet is a risk factor for obesity and steatosis. Reducing intestinal absorption of a high-fat diet (HFD) is a feasible strategy to control obesity. Sulfosuccinimidyl oleate (SSO) inhibits intestinal fatty acid transport. Therefore, the aim of this study was to investigate the effects of SSO on HFD-induced glucose and lipid metabolism in mice and its possible underlying mechanisms.

**Methods:** Male C57/BL were fed a HFD (60% calories) for 12 weeks and were administered an oral dose of SSO (50 mg/kg/day). The expression of lipid absorption genes (CD36, MTTP, and DGAT1) and the serum levels of triglycerides (TGs), total cholesterol (TC), and free fatty acids (FFAs) were detected. Lipid distribution in the liver was detected by oil red and hematoxylin and eosin staining. In addition, serum levels of inflammatory factors, alanine aminotransferase (ALT), and aspartate aminotransferase (AST) were measured to detect side effects.

**Results:** SSO was effective in the treatment of obesity and metabolic syndrome induced by HFD in mice. It attenuated the assembly of intestinal epithelial chylomicrons by inhibiting intestinal epithelial transport and absorption of fatty acids, thereby reducing the gene expression levels of MTTP and DGAT1, resulting in decreased plasma TG and FFA levels. At the same time, it inhibited the transport of fatty acids in the liver and improved the steatosis induced by a HFD. The results of oil red staining showed that SSO treatment can reduce lipid accumulation in the liver by 70%, with no drug-induced liver injury detected on the basis of interleukin-6, C-reactive protein, ALT, and AST levels. In addition, SSO treatment significantly improved insulin resistance, decreased fasting blood glucose levels, and improved glucose tolerance in HFD-fed mice.

**Conclusion:** SSO is effective in the treatment of obesity and metabolic syndrome induced by a HFD in mice. SSO reduces intestinal fatty acid absorption by reducing the inhibition of intestinal CD36 expression, followed by decreased TG and FFA levels, which attenuates HFD-induced fatty liver.

## 1 Introduction

The global epidemic of obesity and its associated metabolic syndrome has stimulated the interest of researchers in the therapeutic outcomes of targeted interventions (Collaborators et al., 2017). Long-term severe obesity can lead to complications, such as type 2 diabetes, steatosis, insulin resistance, and hyperlipidemia (Liu et al., 2017; Liu et al., 2020; Gancheva et al., 2022). Currently, the most effective treatment for obesity is metabolic surgery. In bariatric surgery, gastric bypass and vertical sleeve gastroplasty lead to sustained weight loss through altered body satiety, intestinal nutrient absorption, and endocrine hormones (Cavin et al., 2016; Xie et al., 2019; Anhe et al., 2023). However, people with mild or moderate obesity are resistant to bariatric surgery (Juo et al., 2018; Dolan et al., 2019; Garcia et al., 2022). Thus, pharmacotherapy is accepted as an alternative and promising treatment for many overweight individuals (Alvarez-mon et al., 2021).

Sulfosuccinimidyl oleate (SSO), an inhibitor of fatty acid transporter (CD36), inhibits long-chain fatty acid uptake and glucose uptake in the gut (Baillie et al., 1996; Chien et al., 2011). Recent studies have shown that SSO delays tumor progression by inhibiting lipid transport by tumor cells (Lemberger et al., 2022) and attenuates stroke-induced neuroinflammation (Dhungana et al., 2017). However, few studies have focused on the intestinal lipid absorption phenotype and obesity treatment. The main function of CD36 protein in the intestine (mainly jejunum) is to absorb medium- and long-chain fatty acids, which are transported from the intestinal lumen to intestinal epithelial cells via transmembrane transport. In comparison, absorbed fatty acids are converted into chylomicrons after secondary packaging by MTTP and DGAT1 in intestinal epithelial cells, which subsequently enter the lymphatic system or portal vein (Zhang et al., 2021; Zhou et al., 2022). SSO inhibits CD36. Therefore, we hypothesized that it may affect intestinal lipid absorption through inhibition of the intestinal CD36 expression, thereby improving the metabolic phenotypes induced by lipid excess, such as blood and liver lipid accumulation.

In the present study, we evaluated the effects of SSO on intestinal lipid absorption and the possible underlying mechanisms in mice on a regular diet, high-fat diet (HFD), and leptin-deficient diet. In addition, the therapeutic effects of hyperlipemia [high triglycerides (TGs), total cholesterol (TC), and free fatty acids (FFAs)] and hepatic fat accumulation caused by increased lipid absorption were further investigated.

## 2 Materials and methods

### 2.1 Animal experiments

C57BL mice (20 ± 2 g) and leptin-deficient mice (OB) used in the present study were obtained from the experimental animal center of Gannan Medical College (Ganzhou, China). The animals were maintained at a controlled temperature (22°C ± 1°C), humidity (50%), and light (12-h light/dark). All animal experiments were approved by the experimental animal ethics committee of Gannan Medical College. A HFD consists of 20% carbohydrates, 35% protein, and 45% fat (according to D12492; Research Diets Inc.; 60% of total calories). The SSO drug was obtained from MCE (Hy-112847) and was fed at a dose of 50 mg/kg/day. Blood glucose level was measured using equipment and glucose test strips provided by Sinocare.

### 2.2 Serological tests

After fasting for 12 h, the blood samples from the orbit of mice were collected for serological detection. The fresh blood samples were centrifuged at 6,000 g. Serum levels of C-reactive protein (CRP; H126-1-2), interleukin-6 (IL-6; H007-1-2), aspartate aminotransferase (ALT; C009-2-1), and alanine aminotransferase (AST; C010-2-1) were measured. All reagents were purchased from Nanjing Institute of Biological Engineering, and the test operation was conducted according to the instruction manual. Mouse TGs (F001-1-1), TC (F002-1-1), and FFAs (A042-2-1) were purchased from Nanjing Institute of Biological Engineering and used according to the manufacturer’s instructions. The insulin ELISA test kit purchased from Abcam (AB277390) and used according to the manufacturer’s instructions. HOMA-IR was determined according to the following equation: HOMA-IR = FBG (mmol/L − 1) × fasting insulin (mu/L − 1)/22.5 (Shen et al., 2023).

### 2.3 Glucose tolerance and insulin resistance tests

Before the glucose tolerance test, mice were fasted for 16 h and injected with 2 g/kg of glucose. Blood glucose levels (rat tail blood) were measured at 0, 15, 30, 60, and 120 min after administration. Mice were fasted for 6 h before the insulin resistance test. Blood glucose levels were measured at 0, 15, 30, 60, and 120 min after injection of 0.55 µ/kg.

### 2.4 Real-time fluorescent quantitative RT-PCR

Fresh tissues were grounded and crushed, and the total RNA was extracted using the TRIzol reagent (Ambion). The amplified products were labeled with SYBR (Yeasen; 10222ES60). RT-PCR was performed using the Bio-Rad RT-PCR system. The relative mRNA levels were determined using the comparison threshold cycle method. The primer sequences are presented in Table 1.

**TABLE 1:** Primer information table.

Gene name	Forward	Reverse
Cd36	TTC​CAG​CCA​ATG​CCT​TTG​C	TGG​AGA​TTA​CTT​TTT​CAG​TGC​AG
Mttp	ATA​CAA​GCT​CAC​GTA​CTC​CAC​T	TCT​CTG​TTG​ACC​CGC​ATT​TTC
Dgat1	CTG​ATC​CTG​AGT​AAT​GCA​AGG​TT	TGG​ATG​CAA​TAA​TCA​CGC​ATG​G
Fabp1	GTC​AGA​AAT​CGT​GCA​TGA​AGG​G	GAA​CTC​ATT​GCG​GAC​CAC​TTT
Fatp4	ACT​GTT​CTC​CAA​GCT​AGT​GCT	GAT​GAA​GAC​CCG​GAT​GAA​ACG
Acat	CAG​GAA​GTA​AGA​TGC​CTG​GAA​C	TGC​AGC​AGT​ACC​AAG​TTT​AGT​G
Got2	GGA​CCT​CCA​GAT​CCC​ATC​CT	GGT​TTT​CCG​TTA​TCA​TCC​CGG​TA
Abcg5	AGG​GCC​TCA​CAT​CAA​CAG​AG	GCT​GAC​GCT​GTA​GGA​CAC​AT
Abcg8	CTG​TGG​AAT​GGG​ACT​GTA​CTT​C	GTT​GGA​CTG​ACC​ACT​GTA​GGT
Il-6	CCG​GAG​AGG​AGA​CTT​CAC​AGA	AGA​ATT​GCC​ATT​GCA​CAA​CTC​TT
Tnf-α	GGT​GCC​TAT​GTC​TCA​GCC​TCT​T	GCC​ATA​GAA​CTG​ATG​AGA​GGG​AG

### 2.5 Histological staining

Fresh liver and intestines were fixed with 4% paraformaldehyde for 24 h and embedded with Oct. Then, the samples were sectioned using a Thermo Fisher Scientific slicer with a thickness of 4 µm and set aside at −80°. For hematoxylin and eosin staining, the tissue sections were washed three times with phosphate-buffered saline (PBS), placed in hematoxylin solution for 1 min, washed three times with PBS, placed in eosin solution for 1 min, washed three times with PBS, and sealed with xylene transparent and neutral gum. Photographs of the sections were captured using a Leica microscope. For oil red staining, the tissue sections were washed three times with PBS, stained with oil red solution for 1 min, washed three times with PBS, washed three times with hematoxylin solution for 1 min, washed three times with PBS, and sealed with glycerol gelatin. Photographs of the sections were captured using a Leica microscope. ImageJ software was used to calculate the oil red-stained area.

### 2.6 Western blot analysis

RIPA lysis buffer was used to extract intestinal epithelial tissue protein. BCA protein assay kit was used to determine the protein concentrations (Beyotime, Shanghai, China). The membrane was sealed with 5% BSA for 2 h. The membrane was subsequently compared with the target AMPK-alpha 1 (phospho T183) (1:1,000, AB133448) at 4°C. GAPDH (1:1,000) was used as a loading control. Then, the secondary antibody (1:2,000) was allowed to bind to the primary antibody for 2 h at room temperature. The signal was captured using an emitter-coupled logic substrate (pierce chemical). ImageJ software was used to quantitatively analyze the band intensity.

### 2.7 Statistical analysis

Data are expressed as the mean ± standard error of the mean (SEM). Furthermore, significant differences were determined by performing a *t*-test with least significant difference (LSD) *post hoc* tests, and statistical significance was set at *p* < 0.05.

## 3 Results

### 3.1 SSO could improve the metabolism of glucose and lipid in normal diet-fed mice

**FIGURE 1:**
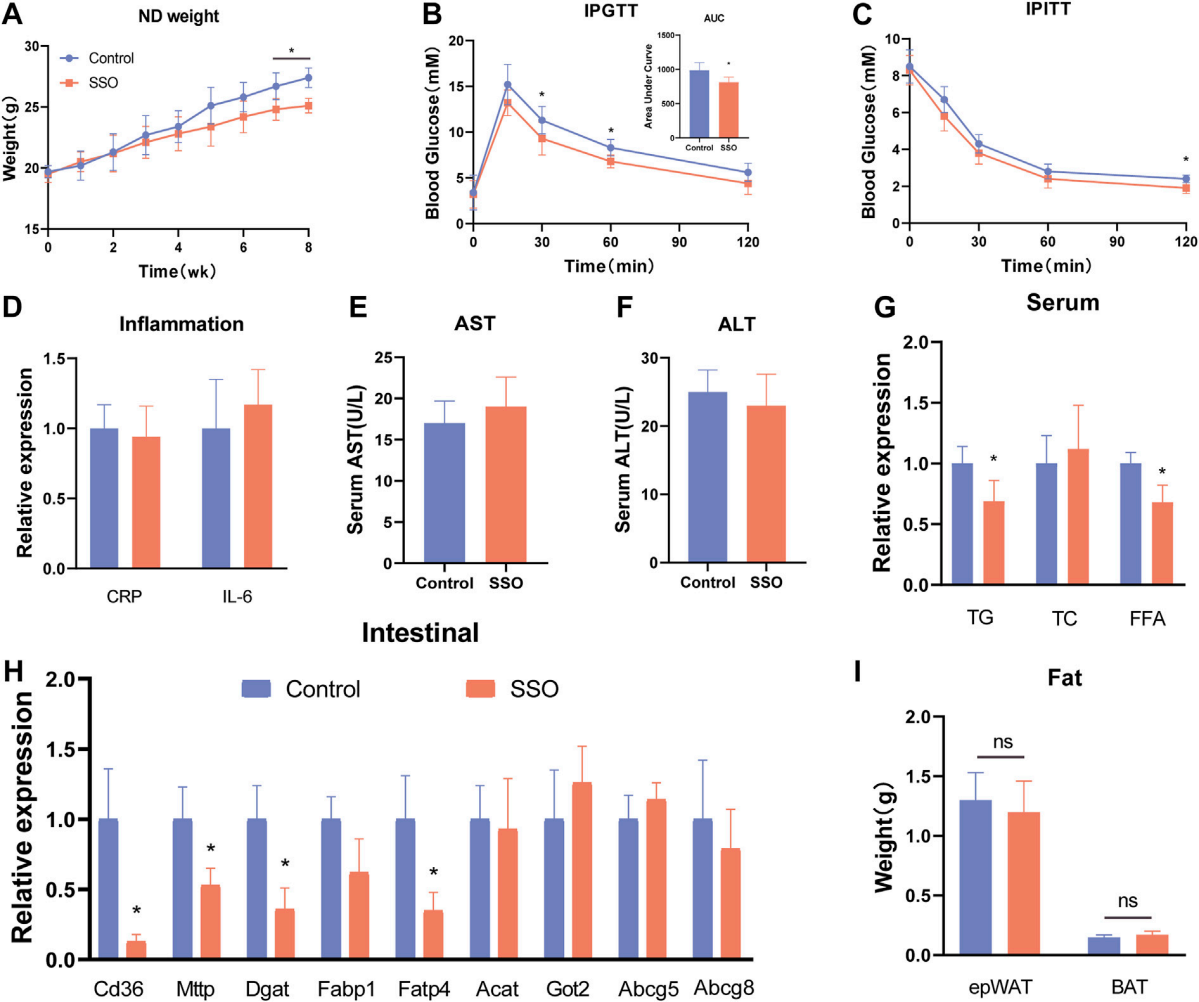
SSO improves the glycolipid metabolic phenotype of mice fed a normal diet. **(A)** Weekly weight gain in mice on a normal diet; **(B)** Glucose tolerance test; **(C)** Insulin sensitivity test; **(D)** Serum CRP and IL-6 expression levels; **(E)** and **(F)** Serum aspartate aminotransferase and alanine aminotransferase levels, **(G)** Serum triglyceride, cholesterol, and free fatty acid levels; **(H)** Intestinal epithelial cell lipid absorption-related gene expression levels; **(I)** Weight of epididymal fat and brown fat. Significant differences are indicated as *p-value <0.05. *n* = 4–6/group.

To explore the effects of SSO on glycolipid metabolism in mice on a regular diet, we fed SSO (50 mg/kg) to mice on a regular diet for 8 weeks, whereas the control group was fed an equal quantity of PBS solvent. The results showed that SSO-fed mice developed weight loss (*p* < 0.05) at 7 weeks compared to the control group (Figure 1A). At the end of the 8-week experiment, we tested the glucose tolerance of mice and found that SSO-fed mice had significantly improved glucose tolerance. In addition, the area under the glucose tolerance test curve suggested improved glucose tolerance in SSO-fed mice (*p* < 0.05) (Figure 1B). Subsequently, insulin sensitivity tests were performed, which showed that SSO-fed mice had significantly increased insulin sensitivity (Figure 1C). To determine the side effects of SSO, we examined the blood levels of CRP, IL-6, ALT, and AST. The results showed that SSO had significant side effects in mice (Figures 1D–F). To further explore the effect of SSO on fat absorption, we measured the blood levels of TG, TC, and FFA. The results showed that SSO-fed mice had lower TG and FFA levels (*p* < 0.05), but no significant change in the TC level (Figure 1G). We examined the expression of genes related to lipid absorption in mouse intestinal epithelial cells to determine the effect of SSO on the gut. The expression of intestinal lipid absorption genes (CD36, MTTP, DGAT1, and FABP4) was significantly decreased in SSO-fed mice (*p* < 0.05), whereas the expression of the reverse lipid excretion gene was not changed (Figure 1H). Furthermore, we did not find any changes in the epididymal fat and brown fat in the SSO mice (Figure 1I).

### 3.2 SSO reduced body weight by decreasing lipid absorption in HFD-fed mice

**FIGURE 2:**
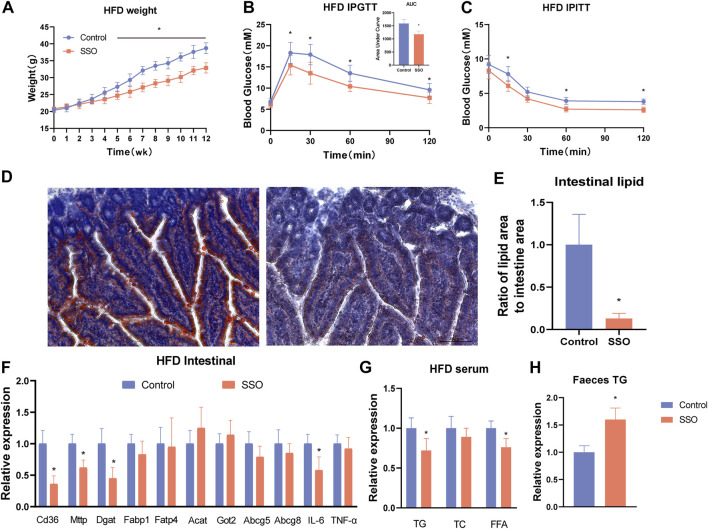
SSO treatment of a HFD-induced glycolipid metabolic phenotype in obese mice. **(A)** Weekly weight gain in mice on a HFD; **(B)** Glucose tolerance test; **(C)** Insulin sensitivity test; **(D)** Jejunum oil red staining of mice on a HFD (20 ×); **(E)** Lipid proportion statistics of jejunum oil red staining in mice on a HFD; **(F)** Lipid absorption- and inflammation-related gene expression levels in intestinal epithelial cells; **(G)** Serum levels of triglyceride, cholesterol, and free fatty acids in mice fed a high-fat diet; **(H)** Fecal triglyceride levels in mice fed a high-fat diet. Significant differences are indicated as *p-value <0.05. *n* = 4–6/group.

To observe the effect of SSO on inhibiting intestinal lipid absorption, we fed SSO to mice on a HFD. The results showed that, during the 12-week observation period, SSO-fed HFD mice had significantly lower body weight than controls at week 5, suggesting that SSO can attenuate HFD-induced weight gain (*p* < 0.05) (Figure 2A). We subsequently performed an intraperitoneal glucose tolerance test (IPGTT) and an intraperitoneal insulin tolerance test (IPITT) test on HFD mice to observe the effects of SSO on glucose tolerance and insulin resistance. We found that, compared to controls, HFD mice fed SSO had significantly improved glucose tolerance and were more sensitive to the hypoglycemic effects of insulin (Figures 2B, C). To directly observe the effect of SSO on the inhibition of intestinal lipid absorption in mice, we sectioned the jejunum of mice on a HFD and stained it with oil red. The results showed that the lipid absorption in the intestine of mice on a HFD was significantly reduced after SSO intake, suggesting that lipids were attached to the lumen. Some lipids were absorbed into the intestinal epithelium. The control group showed a strong ability to absorb lipids, especially for the HFD containing 60% animal fat, which further stimulated the lipid absorption of the intestine. SSO reduced intestinal lipid absorption by 75% (*p* < 0.05) in HFD-fed mice, as measured by the percentage of intestinal oil red staining (Figures 2D, E). These findings suggest that SSO reduces weight gain in HFD-fed mice by inhibiting intestinal lipid absorption.

### 3.3 SSO inhibits lipid absorption by decreasing the intestinal CD36 expression

To explore the inhibitory effect of SSO on intestinal lipid absorption by inhibiting the intestinal fatty acid transporter CD36, we quantified intestinal lipid absorption genes in HFD-fed mice. The results showed that the expression of some lipid genes (CD36, MTTP, and DGAT1) was significantly decreased (*p* < 0.05) (Figure 2F). In addition, to understand the adverse effects of drugs on the gut, we examined the expression of related inflammatory genes and found that SSO did not induce intestinal inflammation. Interestingly, IL-6 expression was lower in SSO-fed HFD mice than in controls (*p* < 0.05). To further explore the reliability of the reduction of intestinal lipid absorption, we measured the levels of TG, TC, and FFA in the blood of HFD-fed mice. The results were consistent with the phenotype of mice fed a normal diet. Compared with the control group, SSO-fed HFD mice had lower levels of TG and FFA in their blood (*p* < 0.05) (Figure 2G). To further investigate the excretion of TG, we examined the fecal TG content in HFD mice. The results showed that the fecal TG content of SSO-fed HFD mice was significantly higher than that of control mice (*p* < 0.05) (Figure 2H). These results suggest that SSO reduces circulating lipid levels by inhibiting intestinal lipid absorption.

### 3.4 SSO treatment reduced fatty degeneration of mice liver induced by HFD

**FIGURE 3:**
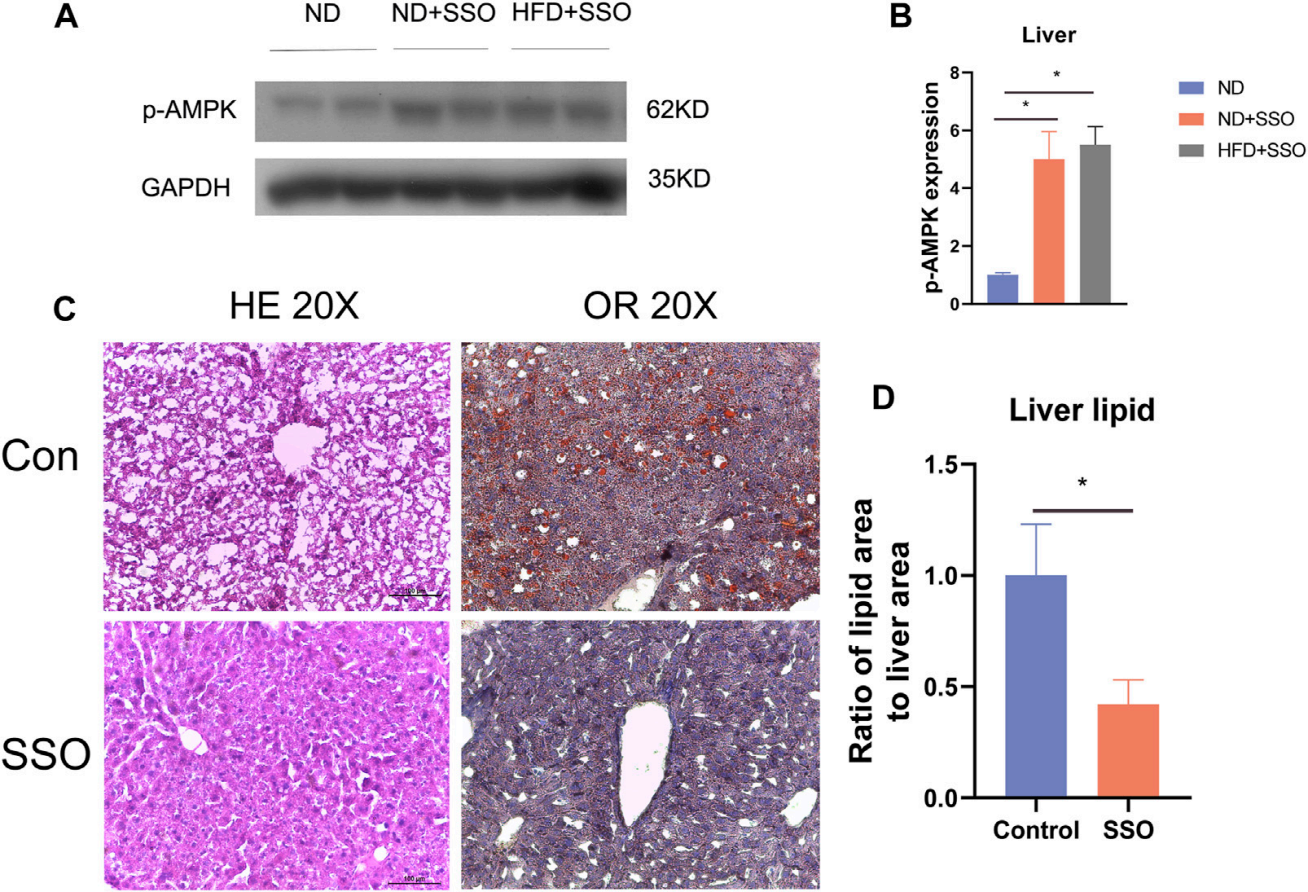
Liver staining results and AMPK protein expression levels in HFD-induced obese mice treated with AMPK. **(A,B)** Western blot plots and statistical plots for AMPK, respectively; **(C,D)** Liver HE staining and oil red staining results and their statistical plots for mice on a HFD. Significant differences are indicated as *p-value <0.05. *n* = 4–6/group.

To confirm the change in the liver AMPK protein expression level, we detected the liver AMPK protein of ND and HFD mice by Western blot. The results showed that SSO-fed ND or HFD mice had a higher AMPK level than controls (*p* < 0.05) (Figures 3A, B). These results suggest that SSO inhibits lipid synthesis by increasing the liver AMPK expression. To further investigate the effect of SSO on lipid accumulation in the liver of mice fed with HFD, liver slices were sectioned and stained with HE and oil red. The results showed that HFD-fed mice, as controls, developed severe lipid accumulation and vacuolar degeneration of the liver. In comparison, SSO-fed HFD mice exhibited significantly ameliorated hepatic steatosis. Quantitative analysis of oil red staining of the liver also showed that SSO attenuated hepatic lipid accumulation in HFD mice (*p* < 0.05) (Figures 3C, D). These results suggest that SSO ameliorates HFD-induced hepatic steatosis.

### 3.5 SSO had no effect on leptin-deficient mice

**FIGURE 4:**
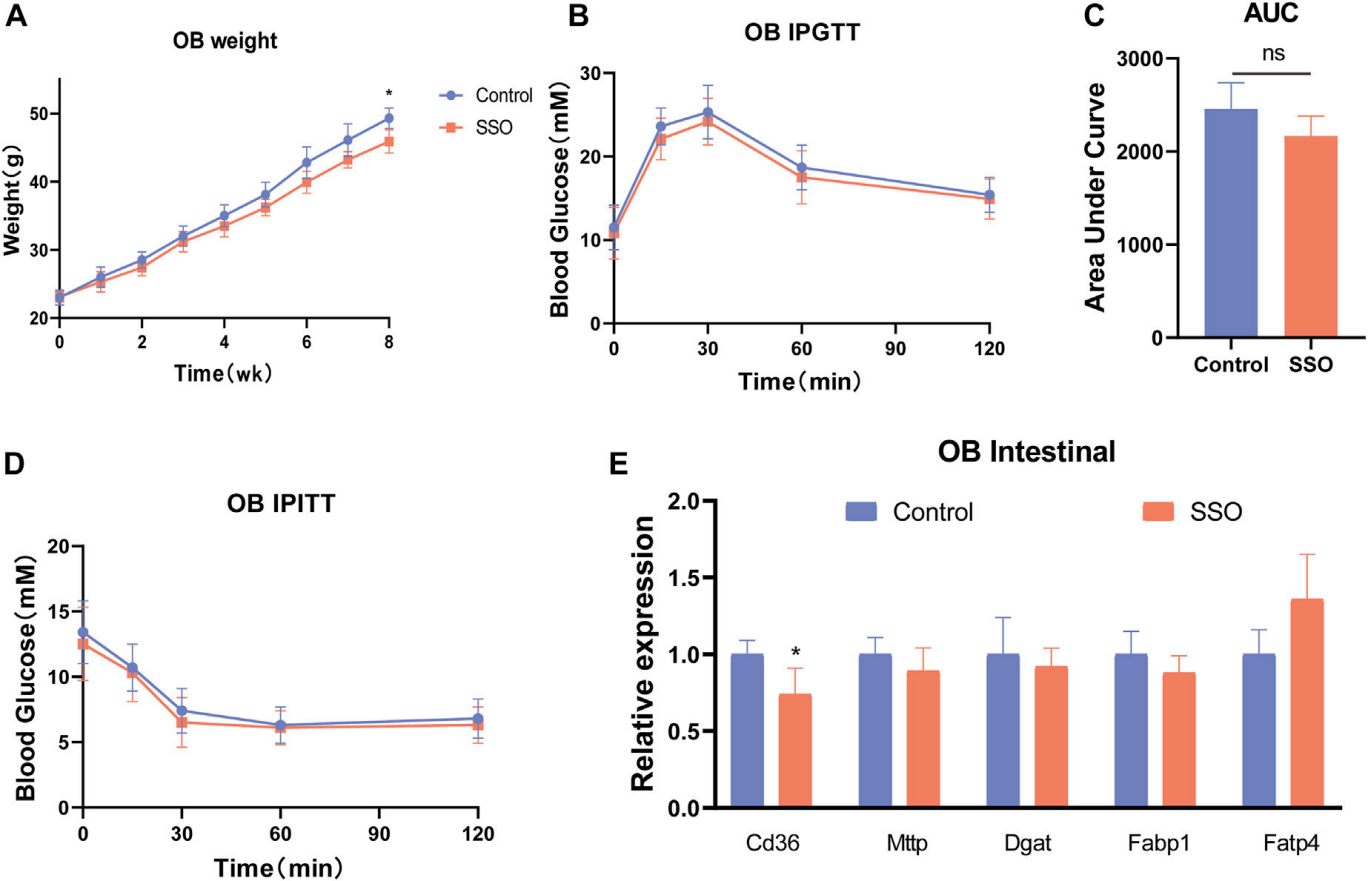
SSO treatment of the glycolipid metabolic phenotype in leptin-deficient mice. **(A)** Weekly weight gain in mice on a normal diet; **(B)** Glucose tolerance test; **(C)** Area under the glucose tolerance test curve statistics; **(D)** Insulin sensitivity test; **(E)** Expression levels of intestinal lipid absorption genes in leptin-deficient mice. Significant differences are indicated as *p-value <0.05. *n* = 4/group.

**FIGURE 5:**
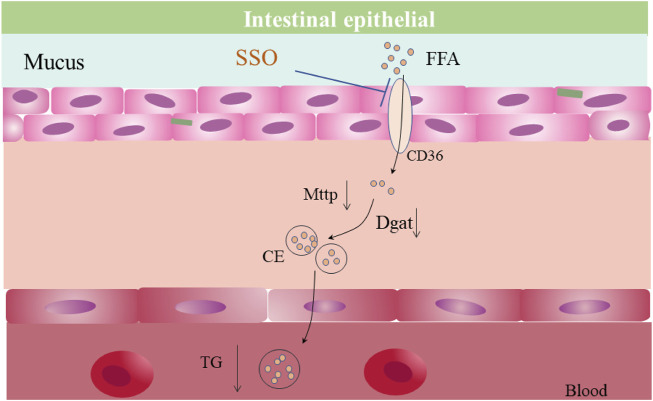
Possible mechanism of reduced intestinal lipid absorption by SSO.

To explore the relationship between the regulation of glycolipid metabolism in mice by SSO and leptin, we used leptin-deficient mice (OB mice) as subjects for SSO feeding. The results showed that SSO-fed OB mice showed a slight weight loss (*p* < 0.05) at week 8 (Figure 4A). However, no differences were observed in the glucose tolerance tests and area under the curve (Figures 4B, C). Next, OB mice were tested for insulin resistance; no therapeutic effect of SSO was found in OB mice (Figure 4D). To further validate the effect of SSO on intestinal lipid absorption genes in OB mice, we dissected the jejunal epithelium of OB mice and examined the associated lipid absorption genes. The results showed that there was no difference in the lipid genes except for CD36 (Figure 4E). These results suggest that SSO has a minor effect on intestinal absorption in OB mice and is not sufficient to ameliorate or compensate for the impaired glucose and lipid metabolism associated with leptin deficiency. Figure 5.

## 4 Discussion

The present results showed that SSO treatment is highly effective in mice with HFD-induced obesity and the related metabolic syndrome. SSO attenuates the assembly of intestinal epithelial chylomicrons by inhibiting the transport and absorption of fatty acids by the intestinal epithelium. It also reduces the gene expression levels of MTTP and DGAT1, resulting in a decrease in plasma TG and FFA levels. In addition, SSO treatment inhibited the gene expression level of CD36 in the liver and ameliorated hepatic steatosis induced by HFS. It also reduced lipid accumulation by 70%, with no evidence of drug-induced liver injury (normal IL-6, CRP, ALT, and AST levels). At the same time, SSO treatment significantly improved insulin resistance, decreased fasting blood glucose levels, and improved glucose tolerance in HFD-fed mice. In addition, SSO was also found to have a small effect on intestinal absorption in leptin-deficient mice, which was not sufficient to ameliorate or compensate for the abnormalities in glycolipid metabolism caused by leptin deficiency.

Globally, the number of obese people is continuously growing. Studies predict that, by 2030, the number of obese people worldwide will reach 1.12 billion (Kelly et al., 2008; Ng et al., 2021). Furthermore, the prevalence of obesity-related metabolic syndromes, such as type 2 diabetes, steatosis, and hyperlipidemia, is also increasing (Luukkonen et al., 2022). A Western diet high in fat and fructose is a risk factor for obesity and the development of non-alcoholic fatty liver disease (NAFLD) (Chen et al., 2023). Therefore, reducing the intake and intestinal absorption of HFD is a feasible strategy for obesity control. HFDs are mostly absorbed by the fatty acid transporter CD36 in the upper jejunum after ingestion by humans and are assembled into chylomicrons by MTTP and DGAT1 within the intestinal epithelial cells. Then, it enters the lymphatic vessels or portal vein through the mediation of apolipoprotein B. CD36 plays an important role in fat absorption.

According to data from 1908 to 1989 in the United States, calorie intake from fat increased from 32% to 45% (Malesza et al., 2021). High intake of fatty acids causes an increase in plasma TG level, which may accumulate in the cytoplasm of hepatocytes in the form of lipid droplets. This, in turn, leads to a fatty liver (Alves-bezerra and Cohen, 2017; Gerges et al., 2021). By reducing the absorption of fatty acids in the intestine and thus causing weight loss, *lactobacillus* (Chung et al., 2016), methotrexate (Fijlstra et al., 2013), piperine (Wang et al., 2021), and *β*-glucan secreted by Rhizobium pusense (Zhang et al., 2022) reduce the intestinal absorption of fatty acids. As a result, there is reduced intestinal absorption of fatty acids and reduction in blood TG level, which ameliorates the diseases caused by TGs (Wang et al., 2021). SSO, an inhibitor of CD36, inhibits cellular uptake of long-chain fatty acids and reduces neuroinflammation after stroke (Chien et al., 2011; Dhungana et al., 2017). However, there are few studies on the intestinal lipid absorption phenotype and the treatment of obesity. Therefore, we hypothesized that SSO might affect intestinal lipid absorption by inhibiting the expression of intestinal CD36, as it can inhibit the absorption of long-chain fatty acids. To test our hypothesis, we used mice fed on a regular diet, a HFD, and leptin-deficient diet. The results showed that mice fed with SSO lost weight on a regular diet, a HFD, and a leptin-deficient diet. In addition, SSO treatment improved glucose tolerance and insulin sensitivity in mice fed a normal diet and a HFD, but not in leptin-deficient mice. Regular diets contain less fat than HFD. HFD contains 45% fat, so SSO has a greater effect on weight loss when mice are fed HFD. A possible reason for our observation is that SSO directly inhibits long-chain fatty acid uptake by intestinal epithelial cells, as suggested by a reduction in lipid absorption from jejunal sections of mice on a HFD (Chien et al., 2011). Weight loss leads to improved glucose tolerance and increased insulin sensitivity (Pareek et al., 2018). To further clarify the effect of SSO on fat absorption, we examined serum lipid content and the expression of genes related to lipid absorption in mouse intestinal epithelial cells in mice on a regular diet and on a HFD. The results showed that the serum concentrations of TG and FFA were lower, but the serum concentration of TC was unchanged. In comparison, the expression of intestinal lipid absorption genes (CD36, MTTP, and DGAT1) was significantly decreased in mice fed with SSO. These results suggest that SSO decreases serum TG and FFA levels by inhibiting fatty acid absorption in the intestinal epithelium.

Serum TG level is closely related to hepatic steatosis (Kaneko et al., 2019; Li et al., 2023). Under normal conditions, the liver stores only a small amount of fatty acids as TGs, whereas under conditions of malnutrition and obesity, elevated serum TG level causes changes in the fatty acid metabolism of the liver. This usually results in the accumulation of intracellular TGs and leads to a clinical condition called NAFLD (Cohen et al., 2011; Alves-Bezerra and Cohen, 2017; Semova and Biddinger, 2021). Therefore, it remains to be explored whether SSO has a therapeutic effect on NAFLD by reducing the serum TG level. To further explore the therapeutic effect of SSO on lipid accumulation in the liver of mice fed a HFD, we sectioned the liver of such mice for HE and oil red staining. The results showed that the control group had severe lipid accumulation and vacuolar degeneration, whereas the HFD mice fed with SSO had significantly improved hepatic steatosis. SSO was also shown to reduce hepatic lipid accumulation in HFD mice. These results suggest that SSO can ameliorate HFD-induced hepatic steatosis.

Some drugs have therapeutic effects on NAFLD, with adverse effects, such as hepatotoxicity (Liu et al., 2022; Geng et al., 2023). To determine the adverse effects of SSO, we detected the serum levels of CRP, IL-6, ALT, and AST. The results showed that CRP, IL-6, ALT, and AST levels did not change significantly after being fed SSO. Because the drug was absorbed directly into the intestine, we examined the expression levels of inflammatory genes (IL-6 and TNF-α) in the intestinal epithelial cells. SSO did not increase the levels of inflammatory genes and reduced the IL-6 expression in the intestinal epithelium of HFD-fed mice. These results suggest that SSO treatment is not associated with hepatotoxicity or other adverse effects.

## 5 Conclusion

SSO is effective in the treatment of obesity and metabolic syndrome induced by a HFD in mice. SSO reduces intestinal fatty acid absorption by reducing the inhibition of intestinal CD36 expression, followed by decreased TG and FFA levels, which attenuates HFD-induced fatty liver.

## Data Availability

The raw data supporting the conclusion of this article will be made available by the authors, without undue reservation.
