# Supplementary figures and images for: Sulfosuccinimidyl oleate ameliorates the high-fat diet-induced obesity syndrome by reducing intestinal and hepatic absorption
Source: Front Pharmacol. 2023 May 26;14:1193006. doi: 10.3389/fphar.2023.1193006 (PMC10254412; doi:10.3389/fphar.2023.1193006)

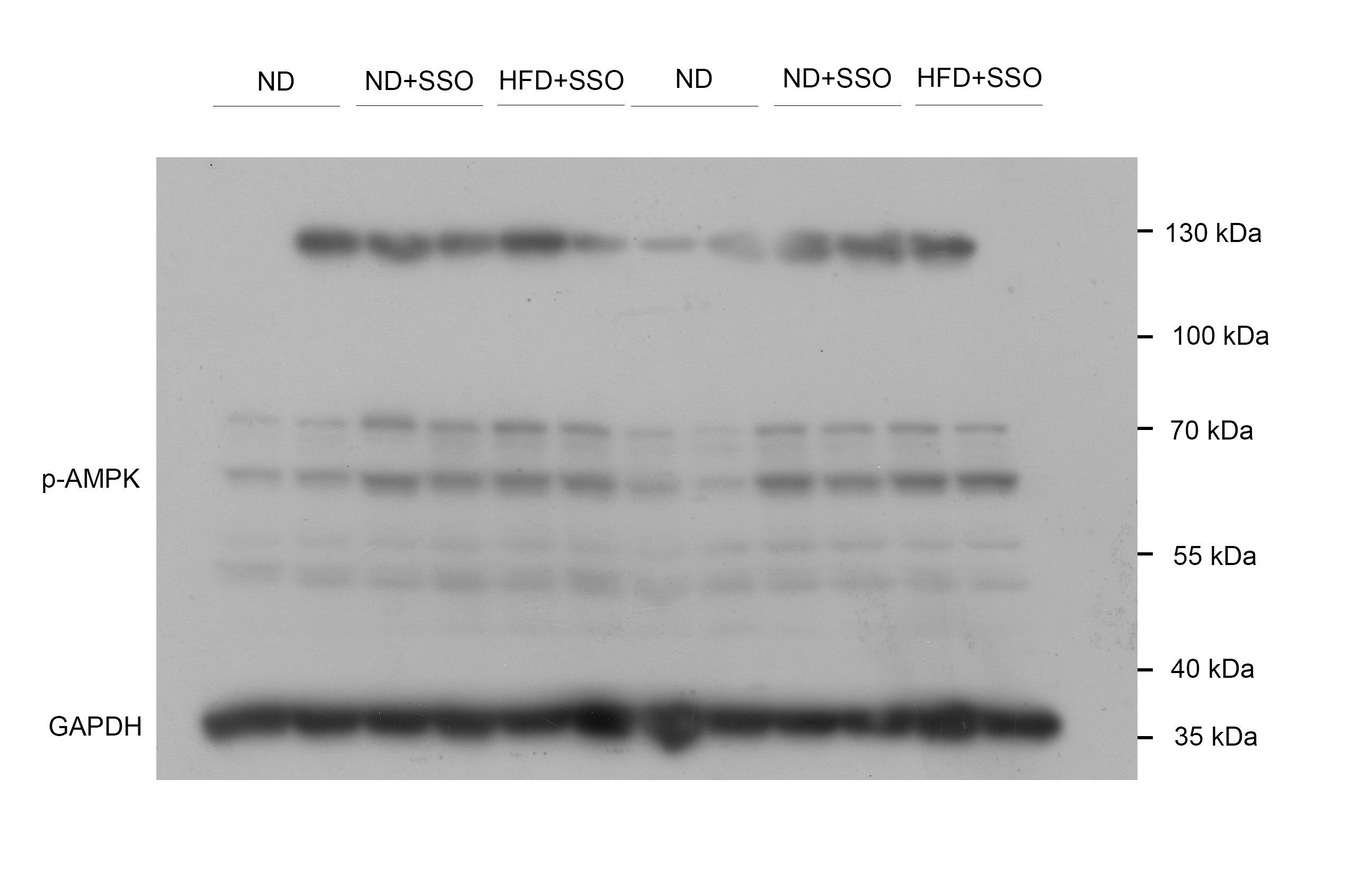

Supplement: Supplementary file 1 [file Image1.TIF]
